# Impact of high prevalence of pseudomonas and polymicrobial gram-negative infections in major sub-/total traumatic amputations on empiric antimicrobial therapy: a retrospective study

**DOI:** 10.1186/1749-7922-9-55

**Published:** 2014-10-25

**Authors:** Moritz T Giesecke, Philipp Schwabe, Florian Wichlas, Andrej Trampuz, Christian Kleber

**Affiliations:** Center for Musculoskeletal Surgery, AG Polytrauma, Charité - Universitätsmedizin, Augustenburger Platz 1, 13353 Berlin, Germany

**Keywords:** Trauma, Open fracture, Amputation, Infection, Pathogen, Pseudomonas, Antimicrobial therapy

## Abstract

**Introduction:**

Emergency treatment of major sub-/total traumatic amputations continue to represent a clinical challenge due to high infection rates and serious handicaps. Effective treatment is based on two columns: surgery and antimicrobial therapy. Detailed identification of pathogen spectrum and epidemiology associated with these injuries is of tremendous importance as it guides the initial empiric antibiotic regimen and prevents adverse septic effents.

**Methods:**

In this retrospective study 51 patients with major traumatic amputations (n = 16) and subtotal amputations (n = 35) treated from 2001 to 2010 in our trauma center were investigated. All patients received emergency surgery, debridement with microbiological testing within 6 h after admission and empircic antimicrobial therapy. Additionally to baseline patient characteristics, the incidence of positive standardized microbiologic testing combined with clinical signs of infection, pathogen spectrum, administered antimicrobial agents and clinical complications were analyzed.

**Results:**

70.6% of the patients (n = 36) acquired wound infection. In 39% wounds were contaminated on day 1, whereas the mean length of duration until first pathogen detection was 9.1 ± 13.4 days after injury. In 37% polymicrobial colonization and 28% Pseudomonas were responsible for wound infections during hospitalization. In 45% the empirc antimicrobial therapy focussed on Gram positive strains did not cover the detected bacteria, according antimicrobial resistogram. It was significantly more often found in infections associated with Pseudomonas (p 0.02) or polymicrobial wound infections.

**Conclusions:**

This epidemiologic study reveals a pathogen shift from Gram-positive to Gram-negative strains with high incidence of Pseudomonas and polymicrobial infections in sub-/total major traumatic amputations. Therefore, empiric antimicrobial treatment historically focussing on Gram-positive strains must be adjusted. We recommend the use of Piperacillin/Tazobactam for these injuries. As soon as possible antimicrobial treatment should be changed from empiric to goal directed therapy according to the microbiological tests and resistogram results.

## Background

The emergency treatment of subtotal and total traumatic amputations (type IIIC open fractures according to the Gustilo/Anderson and type IV according to Tscherne/Oestern classification) with extensive soft tissue injury represents a clinical challenge [1–3]. Although sub-/total traumatic amputations are rare injuries, the potential risk for individual detrimental consequences are serious, including limb loss, infections, osteomyelitis, delayed or non-union, bone segmental defects, soft tissue defects and sensomotor deficits, which in turn cause major socioeconomic costs [4–7].

The successful management of open fractures and traumatic amputations is limited due to high infection rate (<50%), poor soft tissue coverage, impaired fracture healing, non-union and secondary amputation. Traumatic amputations are known to be almost always contaminated with any sort of pathogen, predominately Gram-positive strains [8]. The infection rates are associated with the severity of soft tissue damage (muscle injury, wound contamination, blood supply, reperfusion injury), application of tourniquet in the field and need of fasciotomy in compartment syndrome [1, 2, 6, 9–12]. In particular, sub-/total traumatic amputations with ischemia are more susceptible to infections. Moreover, following vascular repair and reperfusion, severe reperfusion edema and post-ischemic compartment syndrome are observed. Reperfusion syndrome additionally impairs the restored microcirculatory perfusion and local delivery of antibiotics due to endothelial leakage and pre-capillary shunting. Furthermore, traumatic coagulopathy and coagulation factor deficiency (e.g. factor XIII) are known to additionally impair wound healing and predispose towards wound infection [13].

The two cornerstones for successful sub-/total traumatic amputation treatment are emergency surgery with bleeding control, radical debridement, temporary stabilization and early, sufficient empiric antimicrobial therapy [7, 14]. Despite high infection rates up to 66%, the most effective type or combination of empiric antimicrobial agent in open fractures and traumatic amputations are still a controversy [2, 6, 15–18]. In the last decades Staphylococcus aureus, Staphylococcus epidermidis and Gram-negative strains have been the predominate pathogens in these injuries [8]. Empiric antimicrobial therapy is focusing on epidemiologic data, plausible pathogens and patient’s specific comorbidities (e.g. allergy, diabetes). But current epidemiologic data for sub-/total traumatic amputations are not available and microbiological spectrum and corresponding bacterial resistances are changing over the time.

Therefore, this study aimed to retrospectively analyze the characteristics, epidemiologic microbiological pathogen spectrum, incidence of multi-resistant pathogens, the efficiency of antimicrobial therapy, the incidence of multiple trauma and coagulopathy of all sub-/total traumatic amputations, treated in our center.

## Methods

In this retrospective study we included 51 sub-/total traumatic amputations treated at the Campus Virchow Klinikum, Charité - Universitätsmedizin between 2001 and 2010. Due to the retrospective and observational character of the study no approval by our local ethic committee has been necessary.

### Inclusion criteria

-  Major sub-/total traumatic amputation (type IV Gustilo/Anderson Anderson and Tscherne/Oestern; n = 16) [2, 3]
-  Severe open fractures with vascular injury and large soft tissue injury (>5 cm defect or more than loss of 3/4 of circumference; type IIIC Gustilo/Anderson and type IV Tscherne/Oestern; n = 35) [2, 3].

### Exclusion criteria

-  Type IIIc open fractures with <5 cm soft tissue defect or <3/4 of circumference [2]
-  Minor traumatic amputations (finger, toe)
-  Survival <24 hours or patients dying before obtaining microbiological test
-  Lack of substantial information in medical record.

The baseline characteristics (gender, age, trauma mechanism, injury type, anatomical fracture location (AO classification) and following parameters were recorded:

-  Abbreviated Injury Scale (AIS) and Injury Severity Score (ISS)
-  Hanover Polytrauma Score (PTS)
-  Clinical signs of wound infection (rubor, calor, dolor, wound secretion)
-  Microbiological testing (swab, tissue, wound secretion)
-  Pathogen type (Gram positive/negative)
-  Laboratory findings (white blood count (WBC), C-reactive protein (CRP))
-  Type and sufficiency of antimicobial therapy
-  Risk factors for infection (diabetes, immune deficiency, compartment syndrome, arterial occlusive disease, nicotine/alcohol abuse)
-  Length of hospital stay and critical care
-  Type and number of surgical procedures, revisions and use of negative pressure wound therapy (NPWT/VAC)
-  Late complications (osteomyelitis, removal of osteosynthesis materials due to infection, secondary amputation).

The leading injury was defined as the body region (head, face/neck, thorax, abdomen, extremities) with the highest AIS score. Polytrauma was classified according to ISS >15 and severe polytrauma ISS >25.

Traumatic coagulopathy on admission, negatively influencing wound healing, microcirculation and the clinical course of open fractures, was defined by INR >1.2 and/or platelet count <100 000/nl [13, 19].

### Microbiological assessment and definition of wound infections

All microbiological tests of the 51 patients were screened for pathogen type, number of simultaneous pathogens, resistance towards administered antimicrobial agent and time until positive pathogen proof.

Microbiological specimens (i.e. wound swabs, tissue samples, drain fluids) were obtained during surgery before debridement and administration of antibiotic therapy. Within operation at least 2 wound swabs (superficial and deep) were obtained. Based on surgeon’s decision additionally tissue samples were obtain under sterile conditions. Drains were standardized removed within 2 days after surgery except high amount of wound secretion. Therefore, drain fluids were tested in case of persisting secretion >48 hours after surgery. Initial surgical debridement was performed within 2 hours after injury, followed by staged surgical debridement until conditioning of soft tissue or plastic surgery, depending on the clinical situation. Specimens were sent native (i.e. without transport media) and cultivated within 6 hours of sampling, except patient arrived at night, then samples were cultivated next morning. Samples were plated on sheep blood agar plates and incubated for 7 days under aerobic and anaerobic conditions at 37°C; an aliquot was in addition inoculated in thioglycolate broth. Identification and susceptibility testing of grown microorganisms were performed as standard microbiological methods. Wound cultures on day 1 refer to intraoperative microbiological samples collected before the first surgical debridement and antibiotic therapy, reflecting the incidence and spectrum of initial wound contamination.

Wound infection was defined as the proof of at least one pathogenic species in microbiological testing combined with clinical signs of infection (fever, pus, rubor, odor, macerated soft tissue) and elevated laboratory infectious parameter (WBC, CRP). A wound infection occurring within 14 days after trauma was defined as an early infection. Simultaneous detection of more than one pathogen species in the same sample was classified as polymicrobial wound infection. The initially applied antibiotic therapy was considered to be insufficient, if proven so by the microbiological resistance determination via antibiogram.

### Surgical management

Our algorithm for limb salvage and surgical decision making was published in 2010 and performed with the help of the Mangled Extremity Severity Score ≥7, injury severity (polytrauma: “life before limb”) and physiologic parameters (base deficit, lactate, blood pressure, and coagulopathy [6, 20].

Out of 42 cases (82%) of limb salvage, revascularization was successfully performed in 37 cases (73%). In 18% primary (n = 9), 14% (n = 7) macro-replantation and 10% (n = 5) secondary amputation was performed.

Our soft tissue management is guided by the degree of open fracture, contusions, contamination and defect size. According to secondary swelling with progressive soft tissue necrosis and compartment syndrome primary wound closure in type III open fractures was an exception. In our protocol we performed open amputations in case of primary amputation. Criteria for stump formation were clean wean, no pathogen proof obtained in the microbiological test in the prior operation and no need for surgical debridement. We covered the soft tissue defects in type III open fractures with Epigard^®^ + dry dressing in 44 patients, 5 open wound treatment with initial dry dressing and 1 primary NPWT. In 1 case primary wound closure was possible. The dressings were changed at least once every day. NPWT was used in 25 patients (49%) and were changed between 2–5 days according to wound conditions while surgery and results of microbiological testing.

Multiple trauma patients were treated according to their injury severity and hemodynamic constitution with life before limb principle for in extremis patients and damage control orthopedic surgery for hemodynamic unstable patients.

### Statistic

Statistical analysis was conducted by use of PASW statistics 21.0 (IBM, USA; Mann–Whitney-/ Wilcoxon test). Continuous variables were described with standard variation (±) and p-values <0.05 were considered as statistically significant.

## Results

### Characteristics

51 patients were included in this study (male/female: n = 42/9; 82/18%, mean age: 37.5 ± 13.4 years, 62.7% coagulopathy at admission, 7.8% mortality rate). The length of hospitalization in non-survivors (mean 39 ± 42.4 days; range 16–103 days) compared to survivors (mean 39.5 ± 20.4 days; range 7–103 days) showed no statistical significant difference. Therefore, non-survivors were not excluded from analysis.

The mean injury severity was 24.7 ± 13.0 points for ISS and 34.8 ± 20.7 points for PTS. 62% were polytraumatized (n = 31, ISS > 15 points) and 52.9% suffered a severe polytrauma (n = 27, ISS >25 points). The trauma mechanisms are shown in Table 1.

**Figure 1:**
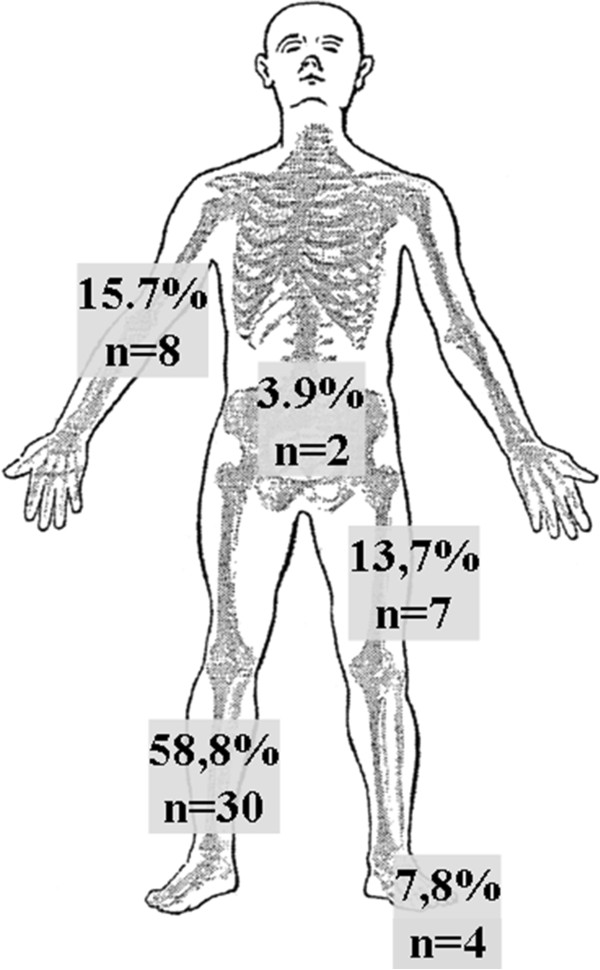
**Anatomic location of sub-/total traumatic amputations.** The lower leg was the predominate injured body region followed by the upper extremity.

In polytraumatized patients the most common injury according to the AIS was extremity trauma (n = 39; 76.5%), followed by chest trauma (n = 9; 17.6%) and abdomen trauma (n = 3; 5.9%). The anatomic location of the open fractures are shown in Figure 1.

**Table 1:** **Trauma mechanism in sub-/total traumatic amputation**

Trauma mechanism								
Injury pattern	Fall	Pedestrian vs. train	Motor-cyclist	Pedestrian vs. auto	Bicyclist	Vehicle occupant	Other	Total
								n
Type IIIC open fracture	11	6	5	6	3	2	2	35
Amputation	1	6	5	2	1	1	0	16
Total	12	12	10	8	4	3	2	51

Distribution of injury pattern according to underlying trauma mechanism in type IIIC open fractures (subtotal amputations) and total traumatic amputations.

### Risk factors for infection

Risk factors for wound infection were negligible (Table 2). We found no significant difference for the rate of wound infections in patient’s with-/out risk factors or/and compartment syndrome.

**Table 2:** **Risk factors for infection**

Risk factor	Incidence	
	n	Total %
Diabetes	1	2
Immune deficiency	0	0
Arterial occlusive disease	1	2
Smoker	3	5.9
Alcohol abuse	1	2
Compartment syndrome	8	15.7

Risk factors in 51 patients with type IIIC open fractures (subtotal-) and traumatic amputations to acquire infection.

### Injuries

68.6% of the patients sustained subtotal (n = 35) and 31.4% total traumatic amputation (n = 16). No statistical significant differences was found for subtotal and total traumatic amputation referring injury severity, hospitalization (39.1 ± 22.4, range 7–103 days), ICU-days (11.9 ± 14.2, range 0–78 days), survival, acute (OF:51%/n = 18; amputation: 69%/n = 11) and total infection rate (OF:66%/n = 23; amputation: 81%/n = 13), type of infection, number and type of needed surgical interventions. Also no differences for the above listed parameters were detected for limb salvage (72.6%; n = 37), primary (17.6%; n = 9) and secondary amputation (9.8%; n = 5). But the mean ISS showed a trend to primary (27.9 ± 12.5 points) and secondary amputation (28.8 ± 15.1 points) compared to limb salvage group (23.3 ± 13 points). Severe polytrauma (ISS > 25) was more frequent in primary (67%) and secondary amputation (60%) compared to limb salvage group (49%).

The number of surgical interventions was 4.4 ± 3.6 for limb salvage, 4.3 ± 3.2 for primary amputation and 9.2 ± 4.4 for secondary amputations. We observed a higher number operations in the secondary compared to the primary amputation and limb salvage group (p 0.04).

The analysis of common laboratory testing (CRP, leucocyte count) revealed no association with the incidence and type of infection.

The traumatic coagulopathy was significantly associated with higher injury severity (PTS p 0.003, ISS p 0.008), severe polytrauma (p 0.02), increased number of surgical revisions (p 0.003) and most relevant, significantly higher infection rate (p 0.03).

In 47.1% (n = 24) late complications occurred (45.8% osteomyelitis, 25% early implant removal due to infection, 29.2% secondary amputation).

### Microbiological findings

In total more than two thirds of the patients (70.6%, n = 36) acquired a wound infections during their hospitalization. The rate of polymicrobial wound infections and Pseudomonas infections in all patients (n = 51) during hospitalization were 37.3% (n = 19) and 27.5% (n = 14), respectively. 14 out of 36 patients with wound infections had a positive Pseudomonas wound swab (38.9%) during hospitalization, in which 50%/n = 7 were detected within 72 hours after admission. The distribution of 14 pseudomonas species have been: 9 P. aeruginosa, 2 P. putida and 1 P. oryzihabitans, P.fluorescens and Aeromonas hydrphilia, respectively.

### First pathogen proof

In 38.9% (n = 14) the first microbiologic test, obtained under sterile conditions within 2 hours after trauma, was positive, indicating that nearly 40% are admitted with contaminated wounds. In contrast, the first positive pathogen proof within several surgical debridement was detected in mean 9.1 ± 13.4 days (range 1–61 days) after injury. Epidemiologic analyses of all pathogen revealed polymicrobial infections (25.5%/n = 13) and Pseudomonas (23.5%/n = 12) as the most frequently detected pathogen in the first positive microbiological testing. Polymicrobial infections assembled in 51.9% from gram-negative and positive strains, 25.9% more than one gram-negative strain and 7.4% more than one gram-positive strain.

### Early infections

In 56.9% (n = 29) patients fulfilled the criteria of an early infection within 14 days after trauma. In 25.5% (n = 13) Pseudomonas species and 23.5% polymicrobial infection (n = 12) were detected in the first positive microbiologic test within 14 days.

**Figure 2:**
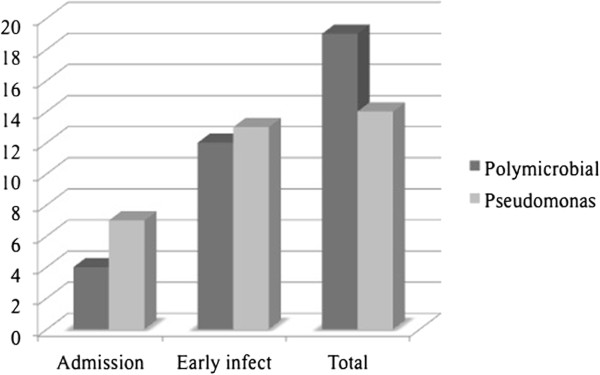
**Temporal incidence of positive polymicrobial and Pseudomonas pathogen proof.** Temporal distribution of positive polymicrobial and Pseudomonas pathogen proof in sub-/total traumatic amputations; y-axis: days after trauma; x-axis: admission: Specimen obtain within emergency surgery; early infect: positive pathogen proof within 14 days after trauma; total: incidence of positive pathogen proof within hospitalization period.

An overview of the epidemiology of pathogens in open fractures and amputations during hospitalization is shown in Table 3 and the temporal distribution of positive pathogen proof in Figure 2.

**Table 3:** **Epidemiology of pathogens in sub-/total traumatic amputation**

		1st wound culture	Acute infection (<14d)	Total
	Pathogen	n	n	n
Bacteria				
Aerobic				
Gram-positive	Staphylococcus aureus	4	2	7
	Bacillus cereus	6	5	6
	Enterococcus faecalis	2	2	5
	Staphylococcus epidermidis	2	2	4
	Enterococcus faecium	3	1	3
	Staphylococcus saprophyticus	1	1	1
	Staphylococcus capitis	0	1	1
	Streptococcus spp.	1	1	1
	Rothia mucilaginosa	1	1	1
	Lactobacillus spp.	1	1	1
Gram-negative	**Pseudomonas spp.**	**12**	**13**	**14**
	E.coli	3	3	5
	Enterobacter spp.	2	2	5
	Acinetobacter spp.	3	3	3
	Pantoea agglomerans	3	3	3
	Stenotrophomonas spp.	0	1	2
	Acromobacter spp.	0	1	2
	Klebsiella spp.	0	1	1
	Proteus spp.	0	0	1
	Chryseomonas luteola	1	1	1
	Acromonas hydrophila	1	1	1
Anaerobic				
	Bacteroides fragiles	1	1	1
	Fusobacterium spp.	1	1	1
	Anaerococcus prevotei	1	1	1
	Clostridium spp.	0	1	1
Fungi				
	Candida spp.	1	0	3
	Aspergillus spp.	1	1	2
	Paecilomyces ilacinus	0	0	1
	Seedosporium spp.	0	0	1
	Mucor spp.	1	1	1
	Fusarium spp.	1	1	1
	**Combined pathogens**	**13**	**12**	**19**
	(>1 species simultaneously)			

Pathogen spectrum of 51 patients with type IIIC open fractures (subtotal-) and traumatic amputations; spp = species. Highest incidence of Pseudomonas spp. and combined pathogens (bolt letters).

Patients with acute Pseudomonas wound infections within 14 days after trauma (n = 13, NPWT 85%, p 0.01) and Pseudomonas wound infections while hospitalization (n = 14, NPWT 79%, p 0.03) were significantly more often treated with negative pressure wound therapy (NPWT) compared to no acute (n = 38, NPWT 37%) or no Pseudomonas wound infection (n = 37, NPWT 38%). We observed no methicillin-resistant Staphylococcus aureus (MRSA) but 13.7% (n = 7) carbapenem-resistant Gram-negative strains. The carbapenem-resistant Gram-negative pathogens were detected at mean 17 ± 10 days (Range 3-31days) after injury.

Neither the incidence of early wound infection nor the fact of completed sufficient initial antibiotic therapy did show a significant effect on the length of critical care treatment and hospitalization period. Also the number of revision surgeries conducted to reach wound closure, soft tissue coverage and skeletal reconstruction did also not significantly differ in patients with early wound infections compared to patients without wound infection. Furthermore, no relation was found between the injury severity and infection rate. Additionally, the injury severity according to ISS and PTS had no significant impact on the incidence of a Pseudomonas or polymicrobial wound infection. We found also no statistical significant differences referring the infection rate, acute infection, Pseudomonas infection, combined infections and appropriate antimicrobial treatment in polytrauma (ISS > 15 points) and severe polytrauma (ISS >25 points) compared to non polytraumatized patients.

### Antimicrobial therapy

All 51 patients received empiric antibiotic therapy after admission. The most common antibiotic agents and frequency of application are shown in Table 4.

**Table 4:** **Antimicrobial therapy**

Antibiotic agent	n	%
Ampicillin/Sulbactam	25	49.0
Ampicillin/Sulbactam + Clindamycin	5	9.8
Amoxycillin/Clavulanic acid	4	7.8
Cefuroxime	3	5.9
Cefuroxime + Clindamycin	2	3.9
Ceftriaxone + Clindamycin	2	3.9
Ciprofloxacin + Clindamycin	2	3.9
Clindamycin	2	3.9

Empiric antimicrobial therapy and frequency of application.

Initial antimicrobial mono-therapy (aminopenicillin plus a beta-lactamase inhibitor) was performed in 66.7% (n = 33) while 29.4% (n = 15) received a double antibiotic therapy. In 45.1% the empiric antibiotic therapy was insufficient according to the later reported microbiological findings and antibiogram. Additionally, the insufficient antibiotic therapy according antibiogram was significantly associated with an increase of Pseudomonas wound infection (<14 days p 0.05; during hospitalization p 0.02).

## Discussion

Open fractures and major traumatic amputations are typically observed among high-energy velocity victims. Appropriate therapeutic management of these severe and rare injuries, including radical debridement, microsurgical revascularization and replantation belongs to the most advanced techniques in orthopedic surgery and requires enormous experience.

### Injuries and coagulopathy

The predominantly injured body region in our collective was the lower leg, which confirms the findings of various other studies [5, 18, 21–27]. Notably, most of the studies exclusively investigated open fractures (type I-III) of the lower leg, whereas our study includes all major traumatic amputations and only type IIIC open fracture within the study period [9, 11, 28–31]. More than half of the patients included in our study were severely polytraumatized (ISS >25 points). We could not find a significant relation between injury severity and infection rate in our study, confirming previously published data for battlefield injuries [12]. We postulate that the individual posttraumatic immune response predispose for infection due to immune depression or pro-inflammatory reactions, not measurable by an anatomical injury severity score [32]. We definitely suggest a specific subgroup of polytrauma patients to be susceptible to acquire infection. Therefore, we need immunological tools to detect and characterize posttraumatic immune response and allocate an individual risk guiding decision-making in polytrauma [32]. In contrast, traumatic coagulopathy was significantly associated with higher infection rate and higher injury severity. Our data suggest that traumatic coagulopathy predisposes for wound infections underscoring the recent detected cross talk of complement system and coagulation factors [33]. We found no statistically significant differences for wound infections in subtotal traumatic amputation and primary major traumatic amputations. It is conceivable, that at this high level of severe soft tissue damage with ischemia in both injury types the already high risk of wound infection does not further increase. Comparison with previous publications is critical, because most studies include all types of open fractures and no traumatic amputation injuries [34–36].

### Microbiology

Due to exclusively type IIIc open fracture the rate of wound infections (70.6%) in our study exceeds the infection rates of previously published data (5-40%) supporting the fact, that the infection rate is strongly related to the extend of the soft tissue injury and vascularization [2, 6, 15, 16, 37].

The epidemiologic part of this study revealed a shift from Gram-positive to Gram-negative pathogens in subtotal and total traumatic amputation compared to literature [1, 8]. We observed a high rate of Pseudomonas and polymicrobial wound infections already at admission to the hospital within emergency operation less then 2 hours after trauma and within hospitalization. This fact indicates the change of natural pathogen environment. Especially Pseudomonas was considered as a late hospitalization infection [12]. We detect a unique high Pseudomonas infection rate of 39% compared to the literature [8, 11, 34]. Furthermore, in our clinical experience we observe more and more primary Gram-negative contaminations or infections also in type II/IIIa/IIIb open fractures. For us, there is a clear trend towards Gram-negative strains in open fractures.

The use of NPWT is a controversy with positive and negative effects [31, 38–40]. In our study we found a significant association between Pseudomonas wound infection and treatment with NPWT. A study revealed the NPWT foam as a hatchery for especially Pseudomonas. Therefore, NPWT has also disadvantages and should not be abused to delay or substitute plastic surgery in traumatic amputations [41]. Temporary soft tissue coverage with artificial skin coverage (Epigard^**®**^) or dry dressings can induce moistening and maceration of the wound. The humid wound atmosphere, fluid collections, bradytrophic tissue in an environment of Gram-positive focused empiric antimicrobial therapy might lead to a survival advantage and secondary selection of Gram-negative pathogens. Additionally the abuse of antimicrobial therapy combined with NPWT has the potential to select multiresistant pathogens, e.g. the carbapenem-resistant Gram-negative strains in our study. All multiple resistances were detected after hospitalization and under antimicrobial therapy at mean 10 days after trauma.

In contrast, the initial temporary vacuum-assisted wound coverage followed by early wound closure or plastic surgery has the potential to lower the secondary infection rates with Gram-negative rods, especially Pseudomonas, because of droughty effects on the wound side and removal of fluid collections predisposed for bacterial secondary colonization with hospitality pathogens in the future.

### Antimicrobial therapy

The change of pathogen spectrum to Gram-negative strains has tremendous impact on the choice of empiric antimicrobial therapy and morbidity of our patients. Former empiric therapy focused on the reported epidemiologic data from 1990s with predominate Gram-positive strains. Therefore, we detect in 45% of our patients the empiric antibiotic regimen as insufficient and were significantly associated with Pseudomonas or polymicrobial wound infections. The data of this study clearly indicate the need to adjust the empiric antimicrobial therapy for type IIIc open fractures and traumatic amputations. However, remarkable controversy exists about the use of antibiotic therapy and guidelines similar to e.g. the 2013 WSES guidelines for intra-abdominal infections are absent [42, 43].

Successful treatment of open fractures and traumatic amputations has two cornerstones, sufficient empiric antimicrobial therapy in combination with immediate radical surgical debridement [1, 6, 10, 15, 18, 31]. We recommend the use of empiric antimicrobial therapy in sub-/total traumatic amputations with Piperacillin/Tazobactam (4.5 g i.v. 3 times per day) [44]. In case of penicillin allergy but no type I allergy (quincke edema, anaphylaxis) we use Cefepim (2 g i.v. 3 times per day). In type I penicillin allergy we use a combination of ether Vancomycin or Daptomycin in combination with Gentamicin or Ciprofloxacin. We always tried to preserve Ciprofloxacin as the only oral Pseudomonas active agent for ambulant long-term treatment of our patients. As soon as available we change from empiric to goal directed antimicrobial treatment according to the microbiological test and resistogram results.

## Conclusions

This epidemiologic study reveals a pathogen shift from Gram-positive to Gram-negative strains with high incidence of Pseudomonas and combined pathogen infections in type IIIC open fractures and major traumatic amputations. Therefore, empiric antimicrobial treatment must be adjusted. We recommend the use of Piperacillin/Tazobactam in sub-/total traumatic amputations. As soon as available antimicrobial treatment must change from empiric to goal directed therapy according to the microbiological test and resistogram results.
